# Supplementary material for: Interfacial engineering of metal-insulator-semiconductor junctions for efficient and stable photoelectrochemical water oxidation
Source: Nat Commun. 2017 Jun 29;8:15968. doi: 10.1038/ncomms15968 (PMC5493770; doi:10.1038/ncomms15968)
Supplement: Supplementary Information [file ncomms15968-s1.pdf]

Type of file: PDF
Size of file: 0 KB
Title of file for HTML: Supplementary Information
Description: Supplementary figures, supplementary notes and supplementary references.

Type of file: PDF
Size of file: 0 KB
Title of file for HTML: Peer review file
Description:

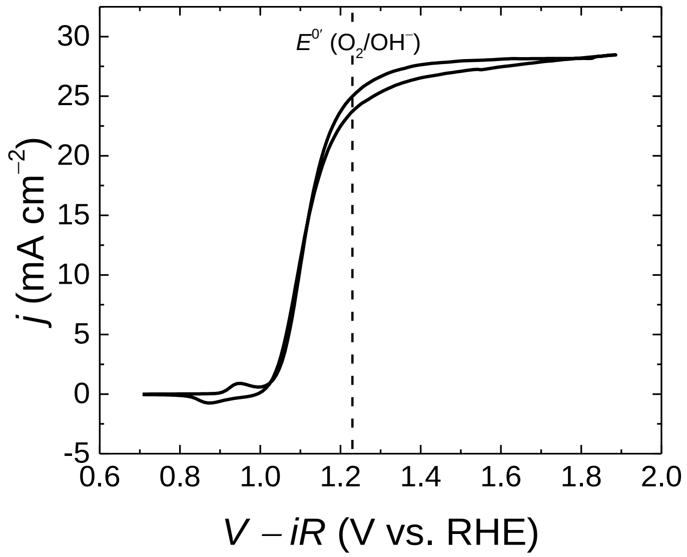

Supplementary Fig. 1 | Corrected cyclic voltammetries of n-Si/SiO_x/Al_2O_3/Pt/Ni photoanode after 18 of aging with compensation for the series resistance. The series resistance of the system was 4.24 Ω cm^2 as measured by EIS, and is consistent with the resistance of 1 M KOH solution.[1]

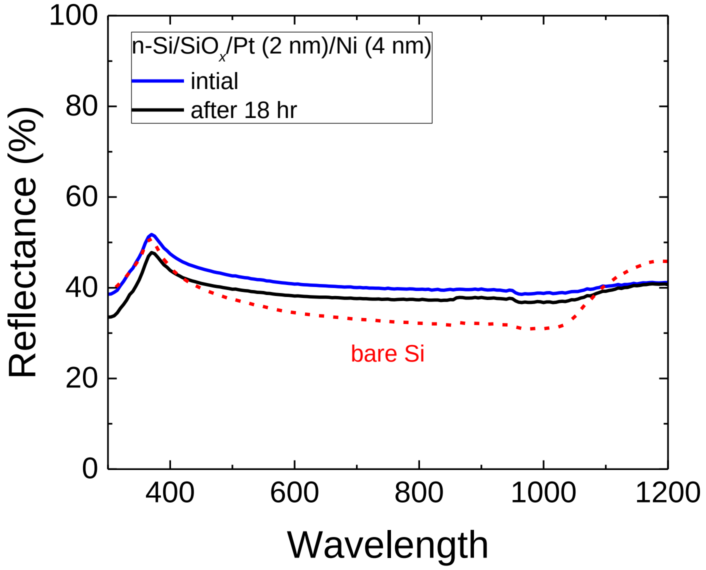

Supplementary Fig. 2 | Reflectance of the fresh and the aged n-Si/SiO_x/Al_2O_3/Pt/Ni, showing a decrease in reflectance in the UV, visible, and near infrared region. For a reference, the reflectance of the bare n-Si/SiO_x (no metal layers on top) is also shown. UV-Vis measurement

was performed using a Perkin-Elmer Lambda 950 spectrometer, equipped with a deuterium and a tungsten lamp to measure the reflectance of the sample in a broad spectral range.

Supplementary note 1

Equivalent photovoltaic response and open-circuit voltage

Equivalent photovoltaic response was determined by defining the photovoltage at each current density as the difference in potential between the photoanode under illumination and the catalyst in the dark.[2,3] Supplementary Fig. 3 shows the photovoltaic response of the n-Si/SiO_x/Al_2O_3/Pt/Ni by comparing its j–V curve under irradiation and the j–V curve of the non-photoactive p^+-Si/SiO_x/ Pt/Ni. The open-circuit voltage (V_{oc}) is defined as the voltage at zero current and the short-circuit density is defined as the current at zero voltage.

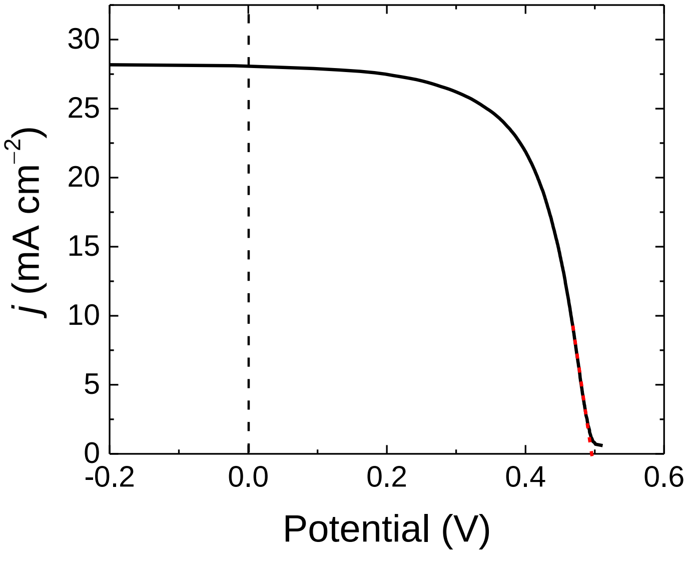

Supplementary Fig. 3 | Equivalent photovoltaic (PV) response of the n-Si/SiO_x/Al_2O_3/Pt/Ni photoanode after 18 agin, showing an open-circuit voltage (V_{oc}) of 0.496 V and a short-circuit current density (j_{sc}) of 28 mA cm^{-2}.

Supplementary note 2

Electrochemical impedance spectroscopy and Mott-Schottky analysis

The EIS data were fit with a model that consisted of a series resistance (R_{series}) in series with a parallel resistor (R_p) and a paralallel capacitor (C_p) to account for the capacitance of the space-charge region, as shown in Supplementary Fig. 4.

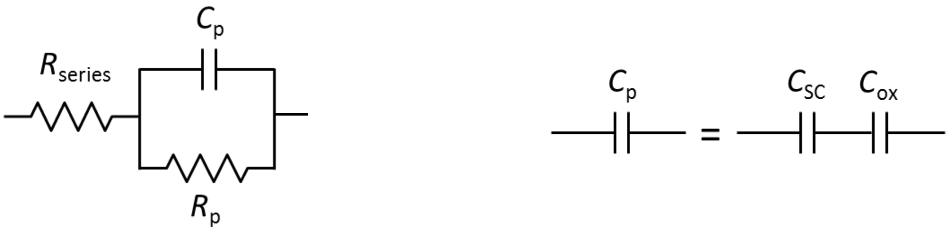

Supplementary Fig. 4 | Equivalent circuit used to fit the EIS data

The C_p is a combination of series contributions that consist of the space-charge capacitance (C_{SC}) and the oxide capacitance (C_{ox}).

$$\frac{1}{C_p} = \frac{1}{C_{SC}} + \frac{1}{C_{ox}} \qquad \text{Eq. 1}$$

The C_{ox} equals to the series capacitance of the SiO_x and Al_2O_3.

$$\frac{1}{C_{ox}} = \frac{1}{C_{SiO_x}} + \frac{1}{C_{Al_2O_3}} \qquad \text{Eq. 2}$$

The capacitances of SiO_x and Al_2O_3 were estimated by the following relationship:

$$C_{SiO_x} = \frac{\varepsilon_0 \varepsilon_{SiO_x}}{t_{SiO_x}} \qquad \text{Eq. 3}$$

$$C_{Al_2O_3} = \frac{\varepsilon_0 \varepsilon_{Al_2O_3}}{t_{Al_2O_3}} \qquad \text{Eq. 4}$$

where the ε_0 is the vacuum permittivity (8.85×10^{-14} F cm^{-1}), $\varepsilon_{\mathrm{SiO}_x}$ is the relative permittivity of SiO_x and is assumed to be the same as SiO_2 (3.9), $\varepsilon_{\mathrm{Al_2O_3}}$ is the relative permittivity of Al_2O_3 (8)[4,5], t_{SiO_x} is the thickness of the SiO_x (1.8 nm) and $t_{\mathrm{Al_2O_3}}$ is the thickness of the Al_2O_3 (1 nm), as measured using the ellipsometer.

Assuming only the space-charge capacitance that varies with the changing electrode potential, the reverse-bias dependence of the inverse square capacitance of the space-charge region in the semiconductor is given by the Mott-Schottky relation:

$$\frac{1}{{C_{\mathrm{SC}}}^2} = \frac{2}{\varepsilon_0 \varepsilon_{\mathrm{Si}} q N_{\mathrm{d}}}\left(V - E_{\mathrm{fb}} - \frac{kT}{q}\right) \qquad \text{Eq. 5}$$

where $\varepsilon_{\mathrm{Si}}$ is the relative permittivity of Si (11.7), q is the elementary charge (1.6×10^{-19} C), N_{d} is the donor concentration in the semiconductor, V is the potential difference between the semiconductor and the redox potential of the solution, E_{fb} is the flat band potential, k is the Boltzmann's constant (1.38×10^{-27} cm^2 kg s^{-2} K^{-1}) and T is temperature (298 K). The E_{fb} was determined by taking the value of the intercept between the extrapolated linear region of the ${C_{\mathrm{SC}}}^{-2}$ with the x-axis in the Mott-Schottky plot.

The barrier height (ϕ_{b}) was calculated using the Schottky's relation:

$$\phi_{\mathrm{b}} = E_{\mathrm{fb}} + V_{\mathrm{n}} \qquad \text{Eq. 6}$$

Where V_{n} is the difference between the potential of the conduction band edge and the Fermi level, and was obtained by using the following relationship:

$$V_{\mathrm{n}} = kT\, ln\left(\frac{N_{\mathrm{c}}}{N_{\mathrm{d}}}\right) \qquad \text{Eq. 7}$$

The density of conduction band states (N_{c}) was calculated by:

$$N_c = 2\left(\frac{2\pi m_e^* kT}{h^2}\right)^{3/2} \quad \text{Eq. 8}$$

where m_e^* is the effective mass of electron in c-Si ($1.08m_0$) and h is the Planck's constant (6.63 Js). Using the slope of Mott-Schottky plot in the main text (3.35×10^{14} F^{-2} cm^4 V^{-1}) and the above equations, the N_d was calculated to be 3.54×10^{16} cm^{-3} and the V_n was calculated to be 0.17 eV. From the calculated N_d the resistivity of the n-type Si wafer was obtained to be 0.185 ohm cm, consistent with the range specified by the Si wafer supplier 0.1-0.3 ohm cm.

The E_{fb} of the fresh and the aged n-Si/$SiO_{x,RCA}$/Al_2O_3/Pt/Ni photoanode were −0.6 and −0.73 V *versus* $Fe(CN)_6^{3-/4-}$, respectively. Using the Schottky relation above, the calculated ϕ_b were 0.77 and 0.9 eV for the fresh and the aged n-Si/$SiO_{x,RCA}$/Al_2O_3/Pt/Ni, respectively.

Supplementary Fig. 5 compares the Mott-Schottky results between the fresh n-Si/$SiO_{x,RCA}$/Al_2O_3/Pt (2 nm)/Ni (4 nm) and n-Si/$SiO_{x,RCA}$/Al_2O_3/Pt (10 nm) without Ni layer. The E_{fb} of both samples was −0.6 V *versus* $Fe(CN)_6^{3-/4-}$ and the slope was $3.35 \pm 0.02 \times 10^{14}$ F^{-2} cm^4 V^{-1}. The same Mott-Schottky results indicate that the effective work function of the Pt (2 nm)/Ni (4 nm) bilayer metals on the fresh sample was not affected by the presence of the Ni surface layer.

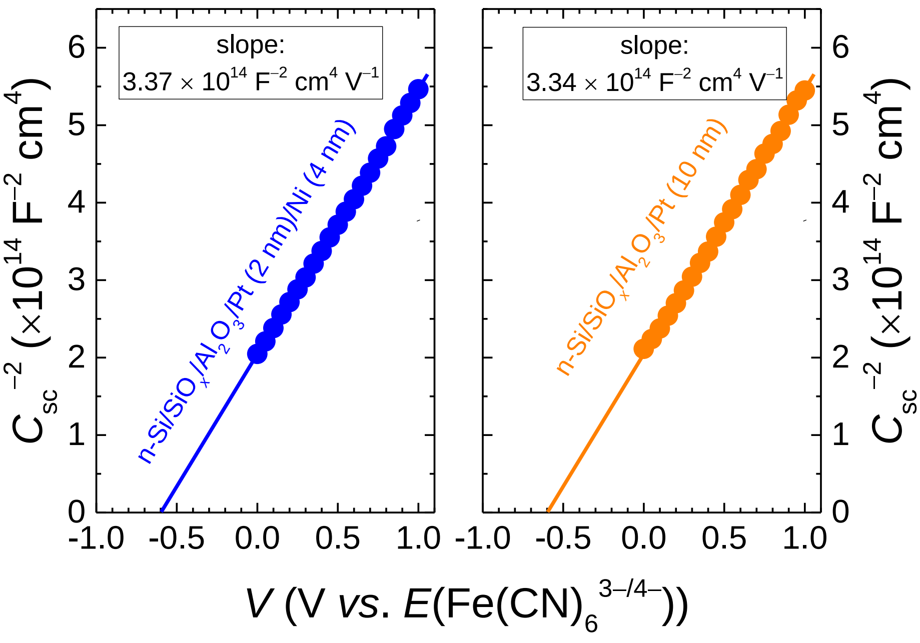

Supplementary Fig. 5 | Mott-Schottky results of n-Si/$SiO_{x,RCA}$/Al_2O_3/Pt (2 nm)/Ni (4 nm) and n-Si/$SiO_{x,RCA}$/Al_2O_3/Pt (10 nm) without Ni layer.

Supplementary note 3

X-ray photoelectron spectroscopy analysis

Nickel oxide surfaces can generally be reduced to its lower oxidation states when using high energy beam during the etching process, and thus may result in an inaccurate analysis. Therefore, the ion etching step should be performed using as low energy as possible. The average etching rate was approximately 3 Å $step^{-1}$ and a total 36 steps of etching were performed on the investigated sample. In the depth profiling analysis, some signals corresponding to different elements overlapped and this is mainly due to the fact that the escape depth of photoelectron is typically 4 nm. Our samples consisted of multilayer stack, and apart from Ni (thickness of 4 nm) and Si (thickness of 525 μm), the rest of each layer has a thickness of less than 4 nm. For example, in our n-Si/$SiO_{x,RCA}$/Al_2O_3/Pt/Ni sample, the thickness

of Pt film was 2 nm and the thickness of the Al_2O_3 was only 1 nm. This means when the ion beam has etched all the Ni away and has reached Pt, the XPS will detect signals not only from Pt but also from Al_2O_3 underneath. The binding energy range for Pt 4*f* is 70-75 eV, the same range as for Al 2*p*. This can possibly obscure the Al 2*p* signals. To avoid this issue, the Al 2*s* was therefore chosen to assign for the Al_2O_3 detection in the depth profiling analysis.

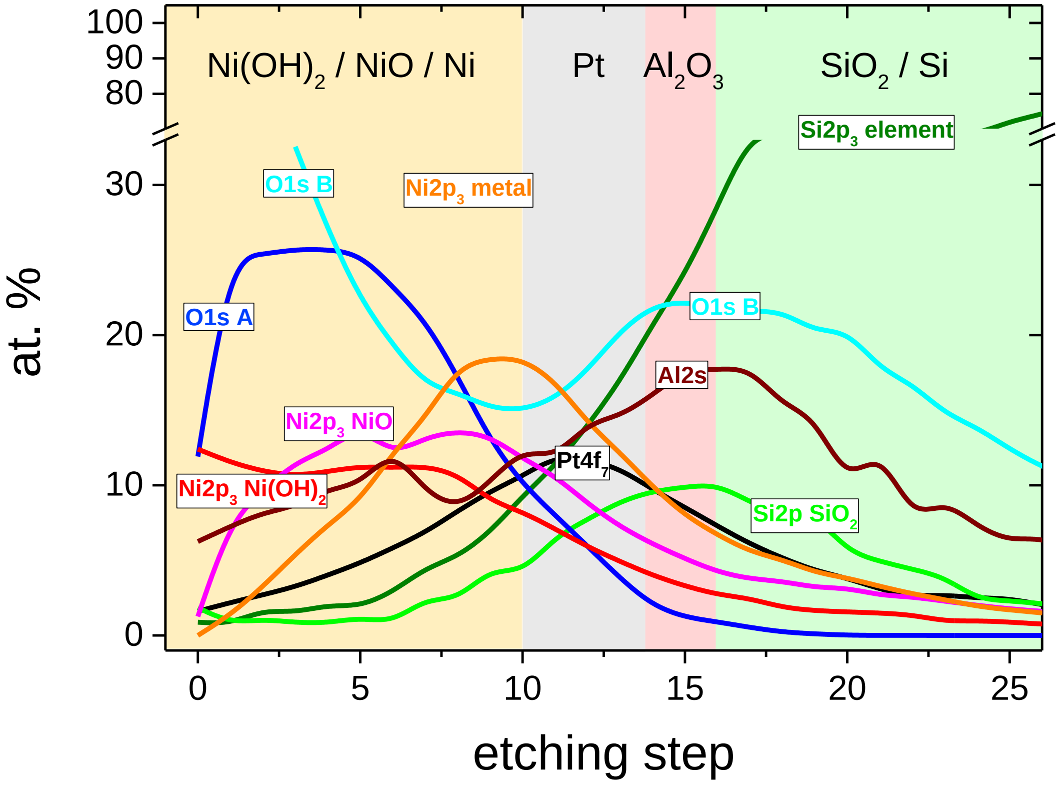

Supplementary Fig. 6 | XPS depth profiling with complete elemental scans of n-Si/$SiO_{x,RCA}$/Al_2O_3/Pt/Ni after 18 hours in contact with KOH solution.

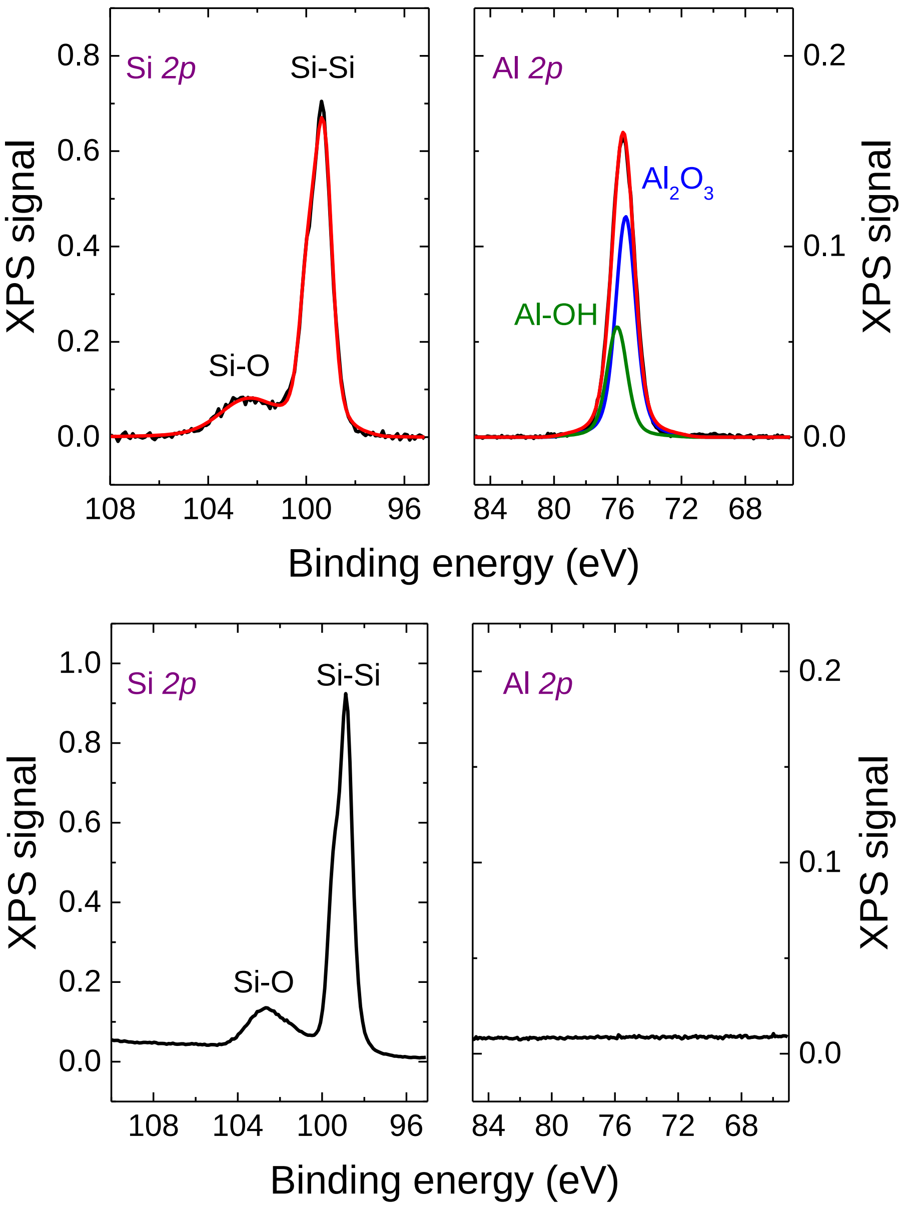

Supplementary Fig. 7 | The XPS spectra of Si/$SiO_{x,RCA}$/Al_2O_3 (1 nm) without Pt and Ni before (top graphs) and after (bottom graphs) immersion in KOH solution for 1 hour. The Al 2*p* signal was completely gone upon contact with KOH, indicating that Al_2O_3 was completely unstable when in a KOH solution.

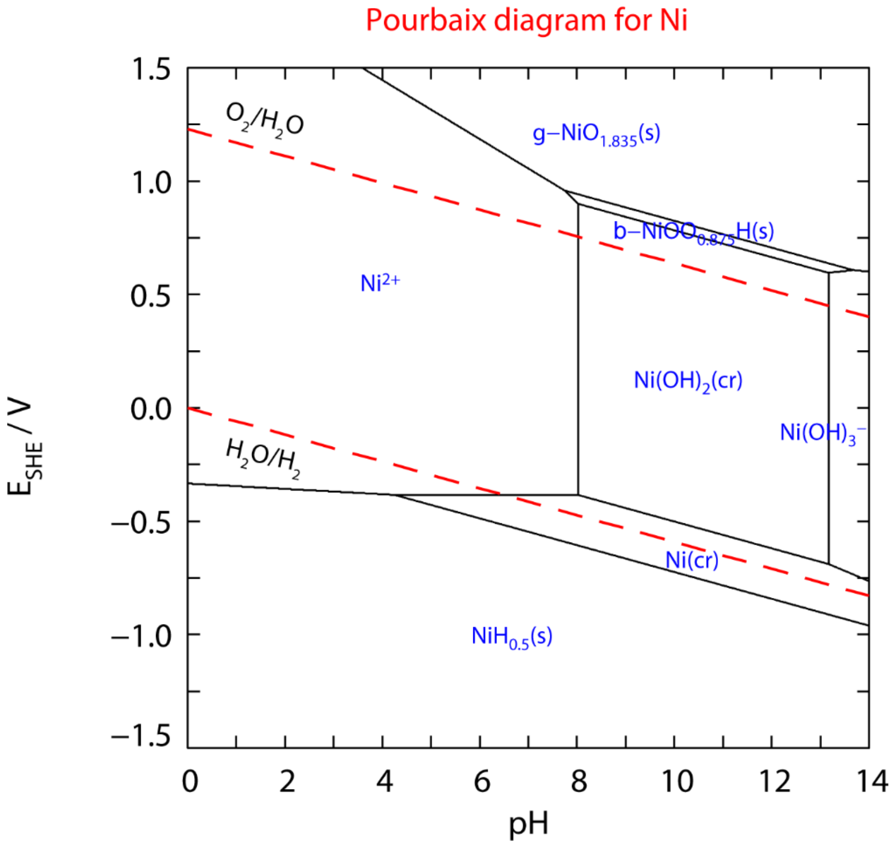

Supplementary Fig. 8 | Pourbaix diagram for Ni.

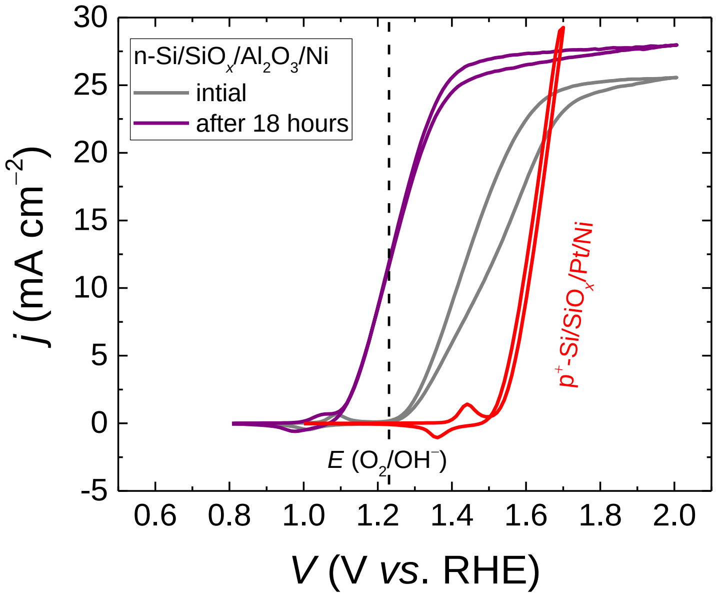

Supplementary Fig. 9 | Initial cyclic voltammetry scans of n-Si/$SiO_{x,RCA}$/Al_2O_3/Ni photoanode (grey curve). The thickness of the Ni was 6 nm. For comparison the voltammetry of the sample after 18 hours of aging in 1 M KOH is also shown (purple curve).

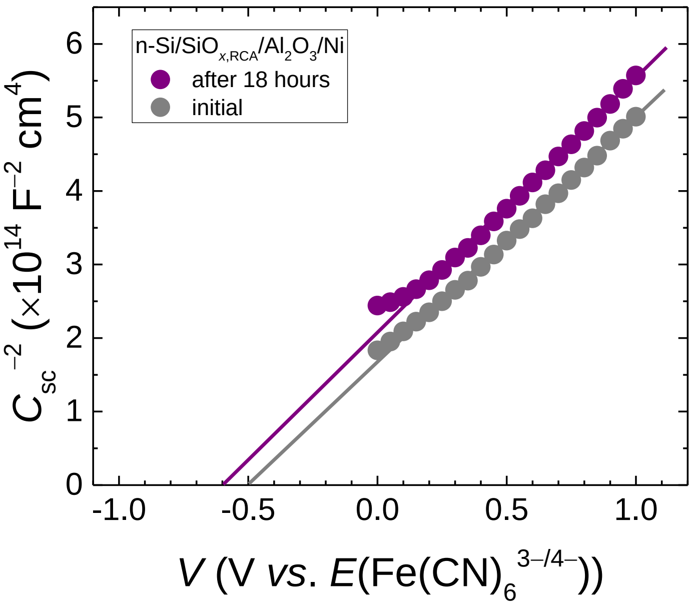

Supplementary Fig. 10 | Mott-Schottky plots of of the fresh n-Si/$SiO_{x,RCA}$/Al_2O_3/Ni and 18 hours of aging treatment in 1 M KOH.

Supplementary References

1. van de Krol, R. & Grätzel, M. *Photoelectrochemical Hydrogen Production*. *Photoelectrochemical Hydrogen Production* **102,** (Springer US, 2012).
2. Hill, J. C., Landers, A. T. & Switzer, J. A. An electrodeposited inhomogeneous metal–insulator–semiconductor junction for efficient photoelectrochemical water oxidation. *Nat. Mater.* **14,** 6751–6755 (2015).
3. Coridan, R. H. *et al.* Methods for comparing the performance of energy-conversion systems for use in solar fuels and solar electricity generation. *Energy Environ. Sci.* **8,** 2886–2901 (2015).
4. Groner, M. D., Fabreguette, F. H., Elam, J. W. & George, S. M. Low-Temperature Al2O3 Atomic Layer Deposition. *Chem. Mater.* **16,** 639–645 (2004).
5. Hemmen, J. L. van *et al.* Plasma and Thermal ALD of Al2O3 in a Commercial 200 mm ALD Reactor. *J. Electrochem. Soc.* **154,** G165–G169 (2007).
